# Editorial: Immune insights into orthopedic infections: mechanisms, biomarkers, and prevention

**DOI:** 10.3389/fcimb.2026.1772866

**Published:** 2026-01-15

**Authors:** Martina Maritati

**Affiliations:** Department of Translational Medicine, University of Ferrara, Ferrara, Italy

**Keywords:** artificial intelligence, biomarkers, biomaterials, immunity, orthopedic infections

Orthopedic infections arise from complex interactions between invading pathogens, the immune system, and the bone–implant interface, resulting in persistent inflammation, biofilm formation, and impaired tissue regeneration. As surgical procedures and implant use continue to rise worldwide, the burden of these infections remains substantial, highlighting the need for more effective diagnostic, preventive, and therapeutic strategies. Their clinical impact extends far beyond the acute infectious episode, influencing tissue healing, implant stability, long-term function, and overall quality of life.

In this context, the Research Topic *“Immune Insights into Orthopedic Infections: Mechanisms, Biomarkers, and Prevention”* was conceived to advance understanding of the immune pathways that shape susceptibility, progression, and outcomes in orthopedic infections; to identify reliable biomarkers for early detection and prognosis; and to promote innovative approaches—including immunomodulatory biomaterials and computational methodologies—that may enhance host defense and ultimately improve clinical care. The contributions collected here collectively expand our knowledge of how immune mechanisms influence musculoskeletal health and how these insights can be translated into more precise and effective interventions.

A series of articles focus on the role of inflammatory biomarkers as tools for refining diagnosis and risk stratification. Tang et al. examine the predictive value of the preoperative C-reactive protein (CRP)-to-albumin ratio and suggest that this simple inflammatory index may help identify patients at heightened risk of developing surgical site infections after kyphoplasty. Their findings align with the growing interest in using immune-derived laboratory parameters to anticipate postoperative complications. In a complementary study, Liu et al. explore the combined diagnostic utility of CRP and procalcitonin in fracture-related infections, demonstrating that the integration of markers reflecting different inflammatory pathways may yield greater diagnostic and prognostic accuracy than single biomarkers alone. The real-world clinical complexity of infection is further illustrated by Qu et al., who investigate culture-negative postoperative infections following percutaneous endoscopic decompression. Their analysis underscores how, in the absence of microbial confirmation, the interpretation of immune-inflammatory indicators and a careful clinical appraisal become fundamental to guide management decisions. Taken together, these contributions highlight how immune-based biomarkers are increasingly central to the early identification and understanding of orthopedic infections.

A second line of work within the Topic addresses how therapeutic interventions can modulate the immune microenvironment. Chang et al. illustrate how vacuum sealing drainage, traditionally viewed as a mechanical strategy for infection control, also influences inflammatory profiles and tissue repair dynamics, ultimately contributing to improved outcomes in secondary bone infections. Their findings reinforce the concept that therapies acting on the local environment inevitably interact with immune pathways. In a broader and more forward-looking perspective, Wang et al. review the expanding landscape of immunomodulatory biomaterials engineered to resist bacterial colonization while shaping host immune responses and promoting osteogenesis. These innovative materials represent a shift toward implant technologies that do not merely tolerate the host environment but actively engage with it to reduce infection risk and improve integration.

An additional and distinctive contribution is offered by Liu et al., who develop an explainable machine-learning model to predict fragility fractures in people living with HIV. Although not focused specifically on orthopedic infections, their work offers valuable insights into how chronic immune dysregulation—characteristic of HIV infection—can compromise bone integrity. By integrating clinical variables into an accessible, explainable web platform, the authors show how artificial intelligence can complement immunological assessments and support personalized risk prediction in populations with altered immune status. Their approach illustrates the potential for data-driven tools to interact with immune-related factors and enrich future strategies in orthopedic medicine.

Finally, Zhang et al. provide a bibliometric overview of research trends in prosthetic joint infections over the past decade, revealing a sustained emphasis on biofilm biology, antimicrobial resistance, and diagnostic innovation. Their analysis reflects a field steadily evolving toward integrated, immune-centered approaches that recognize the central role of host–pathogen interactions.

When considered collectively, these seven contributions offer a coherent narrative in which immunity emerges as a unifying thread across diagnostic, therapeutic, biomaterial, and predictive domains. They demonstrate how inflammation-based biomarkers can refine diagnostic precision, how therapies interact with the immune microenvironment, how innovative biomaterials may prevent infection through combined antimicrobial and immunomodulatory mechanisms, and how machine learning can enhance risk assessment in patients with immune dysfunction. The Topic further illustrates that meaningful progress in addressing orthopedic infections now requires close collaboration among clinicians, immunologists, microbiologists, bioengineers, and computational scientists. Looking ahead, further validation of emerging biomarkers, deeper characterization of immune responses in bone and implant-associated infections, development of dual-function biomaterials, and responsible integration of artificial intelligence into clinical practice will be essential steps toward a more predictive, preventive, and personalized approach to musculoskeletal infection.

**Figure 1:**
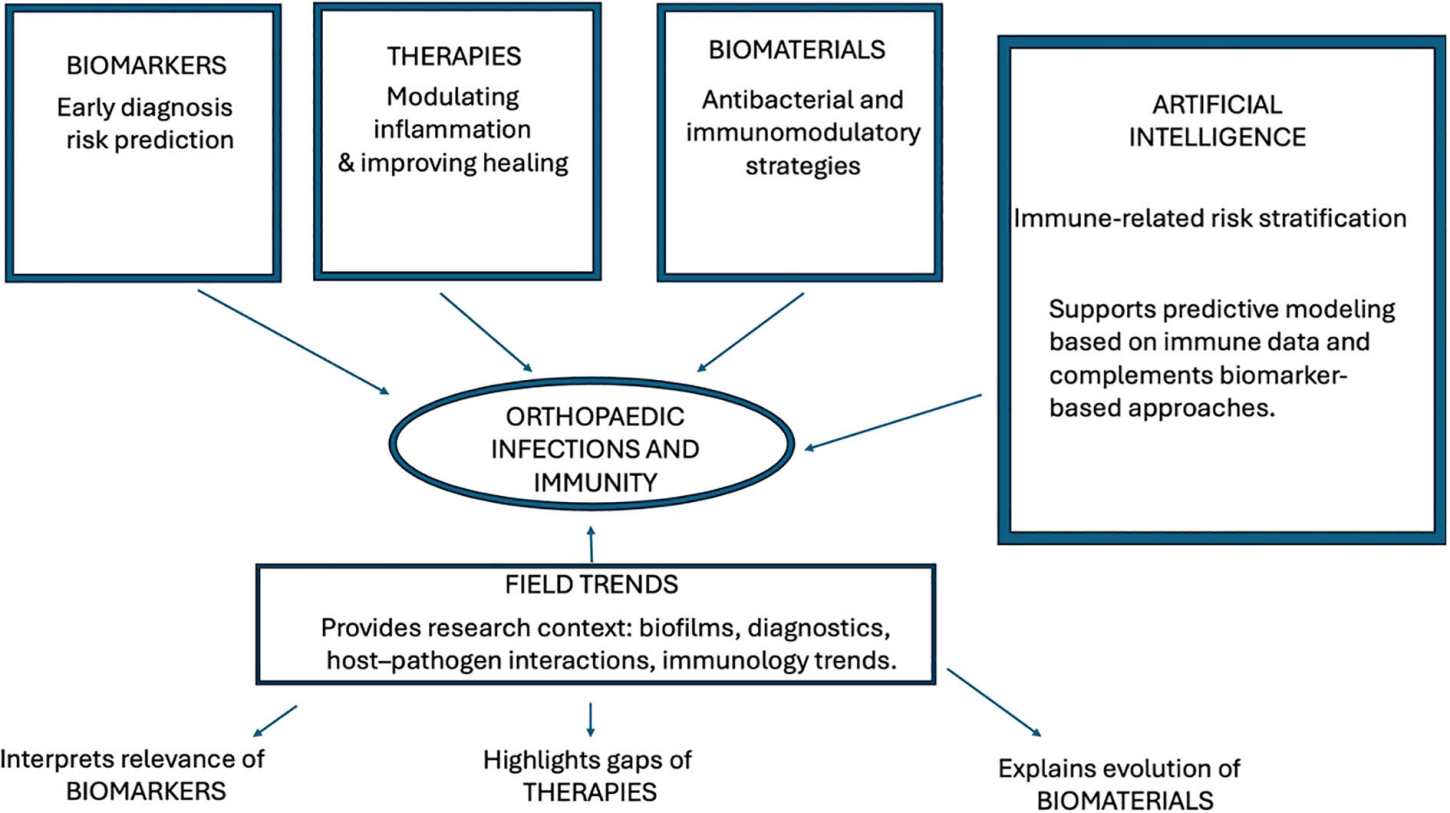
Conceptual map of the Research Topic “*Immune Insights into Orthopedic Infections*.” The central theme—orthopedic infections and immunity—is connected to four major scientific domains represented in the contributing articles: (1) Biomarkers (Liu, Zhang, Li et al.) focusing on early diagnosis and immune-based risk prediction; (2) Therapeutic strategies (Chen et al.) addressing modulation of local inflammation and improved tissue healing; (3) Innovative biomaterials (Wang et al.) integrating antibacterial and immunomodulatory functions; (4) Artificial intelligence and immune-related bone fragility (Liu et al., HIV study) providing personalized risk stratification through explainable machine learning. A bibliometric overview (Zhao et al.) frames these themes within broader research trends, highlighting developments in biofilm biology, diagnostics, and host–pathogen interactions.

In summary, the Research Topic *“Immune Insights into Orthopedic Infections: Mechanisms, Biomarkers, and Prevention”* provides a timely and comprehensive examination of the immunological foundations of orthopedic infection and bone fragility. By bridging basic immunology, clinical investigation, biomaterials innovation, and computational modeling, the topic highlights both the complexity of the challenges ahead and the promise of multidisciplinary solutions (**Figure 1**). We anticipate that these contributions will stimulate further research, strengthen collaboration across diverse scientific fields, and support the development of increasingly effective strategies for preventing and managing orthopedic infections.

